# Functional isoeugenol-modified nanogel coatings for implants

**DOI:** 10.1080/20002297.2017.1325239

**Published:** 2017-06-09

**Authors:** Georg Conrads

**Affiliations:** ^a^ Division of Oral Microbiology and Immunology, Department of Operative Dentistry, Periodontology and Preventive Dentistry, RWTH Aachen University Hospital, Aachen, Germany

## Abstract

With the rapid development of new medical devices including dental implants, bacterial biofilm infections become more and more frequent. We developed a methodology to design bioactive coatings for medical devices based on functional aqueous nanogels. Nanogels with a controlled amount of surface-grafted anti-bacterial molecules (isoeugenol) were synthesized. The crucial step for the nanogel synthesis was the design of hydrophilic macromonomers with covalently attached isoeugenol molecules. This ensured covalent incorporation of isoeugenol into the nanogel surface and effective control over isoeugenol concentration and accessibility. We demonstrated that isoeugenol-modified nanogels exhibit superior antibacterial properties against some prominent pathogens: oral *Streptococcus mutans* and *Porphyromonas gingivalis* as well as *Enterococcus faecalis* and *Staphylococcus aureus*. Nanogels were used as building blocks to design thin functional coatings able to support cell adhesion and osteogenic differentiation of stem cells. These nanogels can be also decorated with RGDs or drug molecules that may accelerate the tissue regeneration process. We believe that the developed nanogel coating technology is a flexible toolbox, which can be used for the decoration of biointerfaces in different medical systems.

